# Nurses’ Experiences in Communicating With Immigrant Populations During COVID-19: Insights and Suggestions for Future Health Crises

**DOI:** 10.1177/10436596241268445

**Published:** 2024-08-02

**Authors:** Seila Mahic, Line Nortvedt, Lise-Merete Alpers

**Affiliations:** 1 VID Specialized University, Oslo, Norway; 2 Oslo Metropolitan University, Norway

**Keywords:** nurses, municipal health service, immigrants, COVID-19, pandemics, information, communication, health literacy, cultural competence

## Abstract

**Introduction:**

When providing health information in a diverse society and during health crises, it is crucial that nurses can adapt their communication to immigrants, as this may have an impact on their health outcomes. This study seeks to identify how nurses experienced and assessed their communication and information work with immigrants during COVID-19 and to discuss measures to improve practice.

**Method:**

The study has an interpretive and explorative qualitative design, analyzing 10 semi-structured interviews with nurses from the municipal health service in Norway.

**Results:**

Three themes were created as follows: multilingual infection control teams and cooperation with volunteers, challenges when providing COVID-19 information, and the nurses’ suggestions for improvements in the event of a new pandemic or other health crisis.

**Discussion:**

Nurses’ cultural competence and their knowledge of immigrants’ health literacy can help them understand how immigrants think and behave during illness. It is important that nurses use discretion during health crises.

## Introduction

In March 2020, the World Health Organization (WHO, 2020b) declared COVID-19 a pandemic, leading to over 700 million cases and nearly 7 million deaths globally (WHO, 2023). Some of the most vulnerable groups during the pandemic were the elderly, children, and immigrants (United Nations [UN], 2020). Health workers worldwide are the main and leading force in the fight against pandemics (Sun et al., 2020). Nurses, as the largest group of health care workers (WHO, 2022), played a crucial role in successful pandemic management, including infection prevention, tracking, vaccination, and patient care (Hafstad, 2021).

To hinder the spread of infection and prevent the disease, Norwegian municipalities took decisive measures. Preventive measures, such as the closing of schools, nurseries, and leisure activities, were implemented to reduce the number of hospitalizations as far as possible and to relieve the burden on the specialist health service (Storhaug & Storli, 2022). In addition, social distancing was enforced, and many workers worked remotely from home offices (NOU, 2021). The WHO (2020a) highlighted, in addition to infection control measures, the importance of being well informed about the disease, as no curative treatment or vaccine was available at the beginning of the virus outbreak.

Nurses, often the first contact in health care (WHO, 2022), had a significant role in communicating with vulnerable populations during the pandemic. Several nurses work in the municipal health service as public health nurses, school nurses, home-care nurses, midwives, and infection control nurses (National Institute of Public Health [NIPH], 2019; Skjøstad et al., 2019). However, most studies that focus on nurses’ experiences during the pandemic put emphasis on nurses who work in hospitals in the intensive care units or other departments with COVID-19 patients. The few studies that have been published on nurses in the municipal health service and their communication and information work about COVID-19 with respect to the vulnerable population often drew attention to nurses working in nursing homes or public schools. For instance, some studies have highlighted how the communication between nurses and residents in nursing homes was challenging during the pandemic due to the use of masks. This is particularly applied to those with impaired hearing who are dependent on lip reading (Diehl et al., 2022; Scerri et al., 2022). Another study reported that school nurses had more health dialogues when offering digital call-in for parents and guardians during the pandemic. It was also highlighted that nurses used prerecorded information videos about the COVID-19 pandemic for students, so they could watch the videos again instead of just hearing the information once (Martinsson et al., 2021).

Despite the fact that several groups in the population were vulnerable to the effects of COVID-19, immigrants were especially overrepresented among infection and hospitalization rates in large parts of the world and throughout the whole pandemic (Norges offentlige utredninger [NOU], 2022; Organization for Economic Co-operation and Development [OECD], 2022). In Norway, this is particularly applied to immigrants with backgrounds from Pakistan, Morocco, Somalia, Iraq, and Turkey (NIPH, 2021). Factors contributing to this include frequent travel, social contact within groups, delayed testing and isolation, and socio-economic status. In addition, other conditions include low health literacy, language barriers, and difficulties in reaching immigrants with effective key messages regarding social distancing, self-isolation, testing, and vaccination (Indseth et al., 2020; Nordic Council of Ministers, 2022).

Norwegian reports confirmed that the health authorities’ information at the start of the pandemic did not reach the immigrant population to the same extent as the rest of the population (Barstad, 2021; NOU, 2022). The failure to reach out had its origin in, among other things, insufficient information and failed communication (Kjeøy et al., 2023). Some people with an immigrant background may also have had difficulties in understanding and applying oral and written information, both from health authorities and health personnel such as nurses. In practice, there is often a large gap between what health care personnel convey and what is actually understood (Safeer & Keenan, 2005). Health literacy has gained attention as one factor related to inequality in health (Ministry of Health and Care Services, 2019) and can be defined as “the ability of an individual to obtain and translate knowledge and information to maintain and improve health in a way that is appropriate to the individual and system context” (Liu et al., 2020). Both studies and reports show that some immigrant groups have lower health literacy compared to the majority population (Le et al., 2021; Wernly et al., 2022).

Increasing cultural competence among nurses and other health care providers is vital for providing equitable health care to diverse populations (Kaihlanen et al., 2019). Today, nurses are expected to be concerned with patients’ perspectives and preferences, and patients are expected to take independent decisions about conditions concerning their health. The former requires communication skills, while the latter presupposes health literacy (Jenum & Pettersen, 2014).

Nurses’ experiences with their communication and information work in respect of certain vulnerable groups during the COVID-19 pandemic have been explored to a certain extent. Nevertheless, there is still a need for research into work aimed at vulnerable immigrants, as, to our knowledge, the existing research is limited. Therefore, the purpose of this study was to fill this research gap by investigating how nurses from the municipal health service experienced and assessed their communication and information work aimed at the immigrant population. We also intend to suggest and discuss measures to improve practice in the event of a similar health crisis.

## Method

### Design

We used a qualitative, interpretive, and explorative design based on hermeneutic methodology. Interpretive description aims to capture themes and patterns within subjective perceptions and generate insights that inform clinical understanding (Thorne et al., 2004). This exploratory approach is suitable when knowledge is limited, seeking new angles and information (Kvale & Brinkmann, 2015). The texts in this study consist of the transcribed interviews. The study followed the Consolidated Criteria for Reporting Qualitative Research (COREQ) (Tong et al., 2007).

### Participants and Recruitment

Participants were recruited using purposive and snowball sampling. Nurses who worked within the municipal health service during the COVID-19 pandemic and had had contact with the immigrant population in Norway were included. The first author (S.M.) contacted the Norwegian Institute of Public Health (NIPH) for assistance from relevant nurses, who were then approached via email. In addition, we used the snowball method. After 10 interviews, data saturation was achieved (Polit & Beck, 2021). Six of the nurses were from Norway, while four of the nurses had backgrounds from Pakistan, Serbia, Somalia, or Turkey. The nurses who worked in the infection control teams were based in Oslo (the capital of Norway); three of the other nurses either worked in the refugee service or in health centers, and two of the nurses were from other municipalities in Norway and worked in health centers (see Table 1).

**Table 1.** Participant Characteristics (n = 10).

Nurse	Gender	Workplace in the municipal health service	1–10 years of work experience	11–20 years of work experience	21–25 years of work experience
1	F	Health center as midwife		X	
2	F	The refugee service	X		
3	F	Infection control team		X	
4	M	Infection control team	X		
5	F	Infection control team	X		
6	M	Infection control team		X	
7	F	Infection control team	X		
8	F	Health center as public health nurse			X
9	F	Health center as public health nurse	X		
10	F	Health center as public health nurse		X	

### Individual In-Depth Interviews

We used individual in-depth interviews to collect data, and collection occurred between January and September 2022. Nine of the 10 interviews were conducted face-to-face via video, as the interviews were performed during the COVID-19 restrictions. The last interview was conducted in person, as the restrictions had been lifted. All the interviews were conducted in Norwegian. The quotes used in section “Results” were translated into English by the first author (S.M.). A semi-structured interview guide was developed to structure the data collection. The topics in the guide were as follows: the role of nurses employed in the municipal health service in connection with the pandemic, their information work targeting immigrants, their experiences and challenges with the information work, and their measures in connection with the information work. The first author (S.M.) conducted the interviews with each participant, and all interviews were audio-recorded and then transcribed verbatim. The interviews lasted from 25 to 61 min (*M* = 35.1 min).

### Data Analysis

Data were analyzed using Graneheim and Lundmans’ (2004) inductive content analysis with both manifest and latent approaches. A manifest approach refers to what the text says and deals with the visible content, while a latent approach involves interpreting the underlying meaning of the text (Graneheim & Lundman, 2004). First, data were read and reread several times, and quotes that contained meaning units related to our research aim were marked and color coded. The quotes that were marked as meaning-bearing units were condensed in the second step of the analysis. In the third step, the condensed meaning units were labeled using codes. Then, in the fourth step, the codes were organized into categories. The final step of the analysis involved merging categories into themes. The heading of a category in inductive content analysis describes the content on a manifest level, with a low degree of interpretation, while a theme is always an interpretation, which involves the content on a latent level (Graneheim et al., 2017). No software was used to manage the data. The analysis was conducted by the first author (S.M.) in collaboration with the other authors (L.N. and L.-M.A.). All the authors are nurses themselves and have worked with immigrants in various settings within the health care system. To ensure the trustworthiness and credibility of the analysis, the authors discussed all the analytic steps until consensus was reached. The analysis process resulted in three themes and five categories, as presented in section “Results.”

### Ethical Considerations

The study was assessed by the Norwegian Agency for Shared Services in Education and Research (Sikt), Reference Number 106529, and conducted following the Declaration of Helsinki principles: informed consent, consequences, and confidentiality (World Medical Association, 2008). An information letter was sent to the participants in advance, and all gave consent to participate in the study. Participants were informed that participation was voluntary and that they could withdraw from the study at any time without reason. The transcriptions were anonymized and stored according to ethical research guidelines, with interview recordings deleted after transcription.

## Results

The study identified three themes: *multilingual infection control teams and cooperation with volunteers*, *challenges when providing COVID-19 information*, and *the nurses’ suggestions for improvements in the event of a new pandemic or other health crisis*. Two of the themes also include specific categories (see Table 2).

**Table 2.** Themes and Categories.

Themes	Category 1	Category 2	Category 3
Multilingual infection control teams and cooperation with volunteers			
Challenges when providing COVID-19 information	Time-consuming and lack of digital equipment	Low health literacy and trust in the authorities	
The nurses’ suggestions for improvements in the event of a new pandemic or other health crisis	Time to prepare information	Organizing of guidelines	Applying the nurses’ interfaces

## Multilingual Infection Control Teams and Cooperation With Volunteers

In order to reach out with COVID-19 information to different immigrant groups, the infection control teams for the Municipality of Oslo recruited nurses who themselves had an immigrant background and were multilingual:

Our teams included people who spoke various languages. We tried to find and hire people who had a medical background and who spoke different languages. (N7)

One of the nurses described this composition of infection control teams at the start of the pandemic:

It was a little randomly organized, but it worked well. It wasn’t the kind of thing that had been tried systematically before, hiring people with minority backgrounds for this work. (N6)

As infection rates in various immigrant groups increased, this approach proved crucial for spreading accurate information to larger sections of the immigrant population. One of the nurses with an immigrant background described her contribution to the dissemination of information:

I worked with contact tracing, mostly by calling those who do not speak Norwegian, and translated the information for them. We also had an information campaign during the quieter periods, by going door to door and handing out more information about vaccines and stuff like that. (N5)

The nurses on the infection control teams also emphasized their cooperation with various resource persons and agencies within the voluntary sector and religious communities:

We used them [the voluntary sector and religious communities] as channels of information, as our partners, working as a team. We explained the measures so that they could spread this knowledge to others. (N7)

Another nurse highlighted the preference for COVID-19 information produced by volunteers rather than the authorities:

it was actually much easier to understand and better linguistically than what the authorities had made, and it was easier to obtain it from the Norwegian Women’s Public Health Association, as there was a shortage of translations. (N4)

The quote emphasizes the importance of available information material in different languages and the value of cooperation with voluntary organizations.

## Challenges When Providing COVID-19 Information

### Time-Consuming and Lack of Digital Equipment

Several of the nurses, particularly the midwives and public health nurses, stated that they did not use the COVID-19 information adapted to immigrants by voluntary organizations or religious communities in consultations, as they did not know it existed. At the same time, several stated how it was time-consuming to find adapted and translated information. One of them said,

You know, during some consultations I tried to find information in their own language, but that was quite difficult. I discovered some shortcuts along the way, but it was not intuitive nor easy to find. (N1)

It turned out that information in languages other than Norwegian on the websites of major health agencies was hidden behind several hyperlinks. The nurse (N1) also explained how consultations could be a challenge when using interpreters because the child health clinics lacked the necessary digital equipment:

We almost exclusively used telephone interpreters during the corona pandemic, and it was much more demanding because things get lost using a phone. At the same time, we don’t really have the equipment for it, so we were stuck with our mobile phones. So, it’s been kind of demanding and makes communication a lot more difficult with those who need an interpreter. The sound was difficult, and remember that everyone was wearing masks, and there are a lot of hurdles to overcome. (N1)

This quote underline how communication between health care personnel, patients, and interpreters is challenging if the technical tools do not work as they should and when physical attendance is not possible.

### Low Health Literacy and Trust in the Authorities

Several of the nurses pointed out that health literacy among the various immigrant groups could vary, and that this was particularly evident in the way they reacted when they learned that they were infected:

. . . understanding health varies among different people and groups . . . I remember a mother who I called to tell that both she and her daughter were infected by corona, and suddenly her daughter picked up the phone and said: “My mom is standing here throwing up.” So, she was so scared that she vomited during that conversation . . . Someone else I called said: “Can’t I just get antibiotics?” So, then you must explain things in detail . . . in such a way that makes them not feel stupid. And always be polite. (N3)

This nurse articulated how a lack of understanding of health can lead to further anxiety during an ongoing health crisis and that it was important to show understanding for the way some people understand health facts and issues. A nurse described encounters with immigrants who did not believe that the viral disease was real but who believed in other theories, and that there was often a lot of uncertainty related to the vaccine:

It was not that they broke the rules set by the authorities, but related to the vaccines and vaccination and that sort of thing . . . They were much more skeptical, and I met a lot of people who believe in conspiracy theories . . . There are also many who believe that the pandemic is not as dangerous as it really is, nor do they fully trust the authorities. The situation with the authorities particularly concerned the migrant workers and those who were new to Norway and perhaps had had some bad experiences. The sort of things they have experienced with individuals representing the authorities, like in asylum centers or stuff like that. (N4)

The quote illustrates how newcomers to Norway with bad experiences with the authorities would trust the authorities less than the rest of the population. The same nurse (N4) also pointed out that some of the information presented by the authorities was not always clear enough, creating confusion:

Among other things, information and recommendations for young women concerning the vaccine. I work with this professionally and understand the information behind it: “If you are planning a pregnancy, then you should not take the vaccine.” But that sentence could actually be interpreted as: “If you’re planning to get pregnant, don’t get vaccinated.” Because if you are a young woman and you are planning to get married and have children in two years, that could easily be misinterpreted. (N4)

Here, the importance of writing and speaking clearly when providing health-related information is emphasized, which can contribute to more accurate and suitable information.

## The Nurses’ Suggestions for Improvements in the Event of a New Pandemic or Other Health Crisis

### Time to Prepare Information

Several of the nurses said that it would have been better if they had been informed of changes to the pandemic measures before they were presented at press conferences. This would have made it easier to provide correct and effective information to both foreign-language speakers and the general population. One of the nurses justified this by saying,

It wasn’t like we were given two hours to prepare, so we watched the government’s press conferences and it was a bit like—“what does that mean?” It wasn’t like NIPH or HelseNorge.no [webpage established by the Norwegian health authorities to convey information to citizens in Norway] had been very good at updating their web pages, because it should have been our “Bible” that we’re supposed to follow, and then people started calling and we had to say, “Sorry, but we don’t know more than you do at the moment,” and I think that’s kind of like a breach of trust. If we only had an explanation of what they [the authorities] were thinking before they addressed the media, perhaps only ten minutes before the press conference, we would have been a few steps ahead . . . (N4)

This nurse underscored the importance of having time to familiarize oneself with new information and new measures, and how late updating of information on the websites of important health agencies delayed this work. Another nurse justified the importance of a system that enables nurses to have time to prepare by saying:

It would have helped to increase the safety of our citizens, by having good and specific answers. (N7)

The quote highlights a crucial aspect of social security and the readiness to have clear responses to different parts of the population.

### Organizing of Guidelines

Some of the nurses expressed a wish for clearer guidelines on how to relate to health institutions in the event of a pandemic. One of them said,

There were some recommendations on NIPH’s website, but healthcare’s chose to make it very different from health clinic to health clinic. So, it’s obvious that there were interpretations. It wasn’t entirely clear. There were discussions like, “How do you do that?,” and you hear that people did it very differently. (N1)

This nurse pointed out that the existing guidelines could be understood in various ways. Better coordination and adaptations to the guidelines can lead to better routines for the nurses’ work and, at the same time, contribute to better knowledge among some immigrant groups on how to relate to the child health clinics during a pandemic. At the same time, another nurse underlined that the health clinic could also have taken more responsibility in preparing suitable information for immigrants:

Of course, we could have made even more general information that was left in the waiting room, like: This is how you should relate to this health clinic. (N10)

The quote illustrates a lack of preparedness aimed at vulnerable groups of immigrants who do not have a good enough command of Norwegian language, but who should be offered targeted information in their own language.

### Applying the Nurses’ Interfaces

Some of the nurses who worked at the health clinics reported that they felt inaccessible when the clinic had to be closed at the height of the pandemic. They pointed to their enormous network of contacts with the parents of children and that they wished they could have been used more to communicate information to parents. One of the nurses recalled,

I felt that the child health clinic was not quite available as a resource. If I remember correctly from research, 98% of parents use the child health clinics for 0–5 years old, and then it is a fantastic opportunity if you could provide some information using us, but I felt that the municipality almost forgot about that opportunity—of using us as a communication channel for parents. But what they are doing in relation to the war in Ukraine right now, is that we have received leaflets and been actively asked to supply brochures to refugees from Ukraine with information. (N9)

This nurse compared how they were part of the information campaign in the context of the ongoing war in Ukraine and said that this way of working was not systematized during the pandemic. She further elaborated,

Nurses are very often given significant trust in what we do, and I think if we had more organized information, I think that would have been good. (N9)

The quote draws attention to the importance of having a system that enables nurses to contribute with more specific information and that they are thus more actively included in situations where they need to reach out with new information.

## Discussion

This study explored how nurses in the municipal health service experienced and assessed their communication and information work with the immigrant population during the COVID-19 pandemic. It also sought their suggestions for improving practices in future health crises.

Some of the nurses in the study considered the inclusion of multicultural and multilingual nurses in infection control teams as an important measure for reaching out with adapted COVID-19 information to various immigrant groups. A Norwegian report described the communication and information from health care personnel in their own mother tongue as a success factor for reaching out to immigrants during the pandemic (Kjeøy et al., 2023). Multilingual and culturally competent nurses are a necessity in today’s health care services (Haqqvist et al., 2020). Their competence can minimize the effects of cultural and ethnic discrimination in care and can give a sense of worth to clients from different cultures (Sharifi et al., 2019).

Several of the nurses experienced low health literacy of some immigrants when providing COVID-19 information. Low health literacy is associated with a limited ability to interpret health information (Jenum & Pettersen, 2014). To deal with complex health information, one needs health literacy to understand, assimilate, and use the information (Choukou et al., 2022). Nielsen-Bohlman et al. (2004) pointed out how inadequate health literacy is considered a silent, hidden epidemic, intertwining a lack of language understanding by the patient with a lack of awareness by health care providers. Within the field of cultural competence, it is emphasized that nurses need knowledge about health-related beliefs and cultural values. Knowledge of such issues helps nurses to understand how the clients think and behave during their illnesses (Campinha-Bacote, 2002). If nurses do not know that health literacy varies among the population and the health services are thus not adapted to different user groups, this may result in different levels of access to health information (Finbråten, Guttersrud, et al., 2023). It is therefore advantageous if nurses use the simplest language possible during consultations, ask control questions to check whether the person has understood, supplement the information with illustrative material if needed (Jenum & Pettersen, 2014), and try to establish trust and security as well as to create a connection with the patient (Finbråten, Romedal, et al., 2023). Such communication strategies can influence large numbers of people in terms of ways to improve their health and health literacy (Elechi & James, 2018).

The nurses in our study demonstrated awareness that the health services could have been better adapted to the immigrant population during the pandemic in terms of even clearer guidelines on how to relate and use the health centers during a pandemic. Jaastad et al. (2020) have argued that some situations can become problematic when laws are read and interpreted too literally by health care personnel. Grimen and Molander (2008) referred to Dworkins’ metaphorical image of professional judgment as a doughnut. The structural side is the ring around the hole and constitutes the restrictions and standards set by the authorities, while the hole in the donut constitutes the free space where the professional, such as a nurse, must assess the situation and make decisions based on discretion. The use of discretion involves applying general knowledge, experience, and judgment to make decisions in certain situations (Grimen & Molander, 2008). Grimen and Molander (2008) asked an important question regarding patient situations where discretion is required: What should weigh more heavily, equal treatment in relation to other patients or the satisfaction of this one patient’s individual needs? The guidelines during COVID-19 were designed to protect people from disease and suffering, but if the guidelines for pandemics or other health crises become too detailed, one can expect that this will impair the nurse’s use of discretion in certain patient situations.

The nurses in our study also stated that health centers could have been better adapted with digital equipment for consultations with interpreters. Mutual understanding is fundamental in interactions between patients and clinicians (The Directorate of Health, 2016). An interpreter, aside from facilitating communication, can help bridge the cultural divide between patients and health care providers (Squires, 2018). Better access to and support for nurses in the use of digital equipment that enables interpretation via screen and mobile phone are therefore of great importance to facilitate adapted communication when the presence of an interpreter is not possible. In addition, it is important for legal reasons, as laws in many parts of the world require health care providers to offer an interpreter if needed.

Some of the nurses had met immigrants who did not believe that the COVID-19 virus was real but rather believed in rumors and conspiracy theories, and several immigrants were also skeptical about the vaccine. Skepticism that the virus was an invention and fear of serious side effects or death as a result of the vaccine were described as very widespread among certain immigrant groups in Norway (Mahic et al., 2023). Our nurses highlighted how especially the information about the vaccines provided by the authorities could be misinterpreted by immigrants due to the way the information was worded. Clarity and some adaptation of information from the authorities to cultural contexts have been demanded by immigrants’ representatives from various groups (Indseth et al., 2021). This may explain why nurses from the infection control teams relied on information from volunteer organizations and religious environments when providing information to immigrants, since they felt that the information was presented in a more understandable language and was easier to find on webpages. However, not all nurses in our study were familiar with the organizations and religious environments providing adapted COVID-19 information. In the event of a new pandemic or other health crisis, we therefore suggest that the municipalities contribute to making it known to more health care workers how to use volunteers and their information work in times of crises. This might reduce duplicate information work and thus facilitate timesaving. In addition, Patient and Public Involvement (PPI) for high-risk individuals and populations during the COVID-19 pandemic can enhance decision-making, risk comprehension, and discussions with nurses, other health care professionals, and family members. PPI might foster diverse community engagement, gives a voice to marginalized populations, combats misinformation, and increases trust in public health measures (Fouladi et al., 2023).

Furthermore, nurses in our study stated that some immigrants showed lower trust in the authorities during the pandemic. This appeared to be particularly heightened among those who have had a short stay and those who had pre-existing reasons for mistrusting authorities or institutions. The nurses in our study believed that this could manifest in lower willingness to get vaccinated. Authorities should, therefore, build trust, as Nichol et al. (2022) have suggested, with the involvement of trusted community leaders and, as several nurses in our study stated, including nurses to an even greater extent when reaching out to different sections of the immigrant population. A Norwegian report referred to high levels of trust in nurses and added that nurses have a good reputation among the population (Ipsos, 2017). Nurses’ involvement may also provide more time for nurses to adapt information to the immigrant population, as they might have better knowledge of the authorities’ next step in handling a pandemic or other health crisis. In addition, Frazar and Davidson (2022) have highlighted how the pandemic has reignited the strategic value and importance of nurses and midwives in planning, developing, delivering, and leading compassionate health systems that are fit for purpose.

## Limitations

This study has some limitations. Using snowball sampling may lead to participants with similar characteristics and interests. To ensure some variation in the data, the nurses from the infection control teams were recruited from different districts in Oslo municipality, and nurses from other municipalities were also included. Second, we continued data collection and interpretation until we reached a point where additional data would only serve to confirm our understanding (Polit & Beck, 2021). However, what constitutes data saturation for one researcher may not be sufficient for another, making it difficult to definitively claim that data saturation has been achieved (Fusch & Ness, 2015). Third, due to the COVID-19 measures, most of the in-depth interviews were conducted digitally. In a few cases, this presented challenges at the start of some interviews related to the clarity of voice and image. This caused short disturbances that would not have occurred otherwise. However, the interviews were not initiated until such disturbances had been resolved.

## Conclusion

The inclusion of multilingual and multicultural nurses in infection control teams is essential. Providing information in immigrants’ mother tongues was effective during the pandemic. Understanding immigrants’ health-related beliefs, cultural values, and health literacy is crucial for effective communication. Municipalities should promote the use of volunteers and their information efforts during crisis.

These findings can guide municipalities, health authorities, and health care leaders in supporting nurses working with vulnerable groups during pandemics and other health crises.
